# A Bio‐Based Supramolecular Adhesive: Ultra‐High Adhesion Strengths at both Ambient and Cryogenic Temperatures and Excellent Multi‐Reusability

**DOI:** 10.1002/advs.202203182

**Published:** 2022-08-09

**Authors:** Peng Sun, Shan Mei, Jiang‐Fei Xu, Xi Zhang

**Affiliations:** ^1^ Key Laboratory of Organic Optoelectronics & Molecular Engineering Department of Chemistry Tsinghua University Beijing 100084 P. R. China

**Keywords:** bio‐based materials, cryogenic adhesives, reusable adhesives, supramolecular polymer materials

## Abstract

Developing high‐performance and reusable adhesives from renewable feedstocks is of significance to sustainable development, yet it still remains a formidable task. Herein, castor oil, melevodopa, and iron ions are used as building blocks to construct a novel bio‐based supramolecular adhesive (BSA) with outstanding adhesion performances. It is prepared through partial coordination between melevodopa functionalized castor oil and Fe^3+^ ions. Noncovalent interactions between adherends and the catechol unit from melevodopa contribute to reinforcing adhesion, and the metal‐ligand coordination between catechol and Fe^3+^ ions is utilized to strengthen cohesion. By combining strong adhesion and tough cohesion, the prepared BSA achieves an adhesion strength of 14.6 MPa at ambient temperature, a record‐high value among reported bio‐based adhesives as well as supramolecular adhesives to the best of knowledge. It also outperforms those adhesives at cryogenic temperature, realizing another record‐high adhesion strength of 9.5 MPa at −196 °C. In addition, the BSA displays excellent multi‐reusability with more than 87% of the original adhesion strength remaining even after reuse for ten times. It is highly anticipated that this line of research will provide a new insight into designing bio‐based adhesives with outstanding adhesion performances and excellent multi‐reusability.

## Graphical abstract

An ultra‐strong and multi‐reusable bio‐based adhesive is successfully constructed by utilizing castor oil, melevodopa, and iron ions as building blocks. By combining strong adhesion with tough cohesion, it achieves record‐high adhesion strengths of 14.6 MPa at ambient temperature and 9.5 MPa at −196 °C. What's more, it exhibits excellent multi‐reusability of ten times with over 87% adhesion strength remaining.

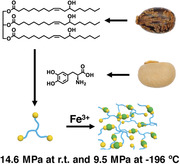

## 1 Introduction

Sustainability is the cornerstone of future chemical sector and of crucial importance to human society due to petroleum supply shortages and environmental pollution.^[^ ^1^, ^2^, ^3^, ^4^, ^5^ ^]^ Among promising solutions to it, constructing environmentally friendly materials from renewable feedstocks has attracted broad attention in both industry and research.^[^ ^6^, ^7^, ^8^, ^9^, ^10^ ^]^ The field of adhesives is also witnessing this evolution by increasing endeavor that has been devoted to developing bio‐based adhesives from plant oils, proteins, sugars, etc.^[^ ^11^, ^12^, ^13^, ^14^, ^15^, ^16^, ^17^ ^]^ Interestingly, the concept of bio‐based adhesives is not new, and it can date back to the ancient age, when one utilized natural macromolecules from plants or animals to make glues. In the twentieth century, with the boom of polymer science, petroleum‐derived adhesives replaced natural glues and dominated the global market gradually on account of their improved bonding strength and satisfactory water resistance.^[^ ^14^, ^15^ ^]^ Meanwhile, it remains a formidable task to develop high‐performance bio‐based adhesives, with performances even superior to their petroleum‐derived counterparts.

Tremendous efforts have been devoted to enhancing the bonding strength of bio‐based adhesives, and those design notions can be divided into two categories: reinforcing adhesion and strengthening cohesion.^[^ ^14^, ^15^, ^18^, ^19^, ^20^ ^]^ Adhesion refers to the interfacial strength between adhesives and adherends. Strong adhesives are always found to adhere to adherends tightly through noncovalent interactions such as hydrogen bonding, metal‐ligand coordination, host‐guest interaction, etc.^[^ ^21^, ^22^, ^23^, ^24^, ^25^, ^26^, ^27^ ^]^ Cohesion is defined as the internal strength of adhesives. Adhesives with tough cohesion have the capability of transmitting heavy loads between adherends without failure.^[^ ^28^, ^29^, ^30^, ^31^, ^32^, ^33^ ^]^ Apart from being utilized to reinforce their adhesion, noncovalent interactions are often employed to strengthen adhesives’ cohesion by enhancing the interaction among polymer chains or constructing supramolecular polymer networks.^[^ ^34^, ^35^, ^36^, ^37^ ^]^ In other words, noncovalent interactions can spontaneously strengthen adhesion and cohesion. Impressively, by exploiting hydrogen bonding between crown ether and water molecules, strong adhesives with outstanding resistance to the extremely low temperature can be developed.^[^ ^38^ ^]^ Therefore, we wonder if we could design a high‐performance bio‐based adhesive by constructing a partially crosslinked supramolecular network. These noncovalent interactions could further endow bio‐based adhesives with desirable reusability, which may contribute to extending the service life and cost saving.^[^ ^31^, ^39^, ^40^, ^41^, ^42^ ^]^

**Scheme 1:**
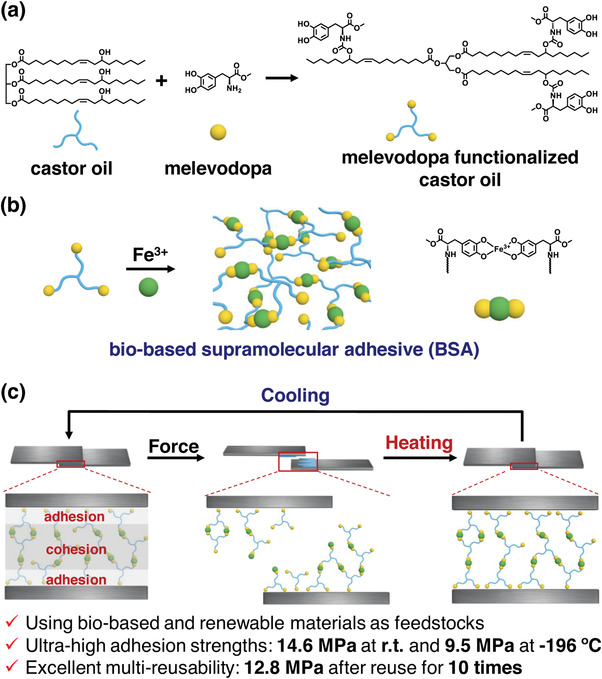
Schematic diagrams of a) the synthesis of melevodopa functionalized castor oil, b) the formation of BSA, and c) the robust adhesion strength and multi‐reusability of BSA.

Herein, we report a novel bio‐based supramolecular adhesive (BSA) with outstanding bonding performances by using castor oil, melevodopa, and iron ions as building blocks. It is revealed that the catechol unit is capable of forming various noncovalent bonds with different materials.^[^ ^21^, ^26^, ^43^, ^44^, ^45^ ^]^ Meanwhile, the catechol unit is also found to have the capability of coordinating with diverse metal ions to form bis‐coordinated catechol‐metal ion bonds.^[^ ^46^, ^47^, ^48^, ^49^, ^50^, ^51^ ^]^ As shown in **Scheme** **1**a,b, we designed and synthesized a three‐arm molecule, whose skeleton stemmed from castor oil, and each of its arms was functionalized with melevodopa. Then the BSA was prepared through partial coordination between melevodopa functionalized castor oil and Fe^3+^ ions. Castor oil is extracted from castor seeds directly and melevodopa can be synthesized from levodopa collected from *Mucuna pruriens* seeds, meaning that the BSA utilizes bio‐based and renewable materials as feedstocks. As illustrated in Scheme 1c, the BSA could be endowed with strong adhesion through noncovalent interactions between adherends and the catechol unit from melevodopa, and tough cohesion through the noncovalent crosslinking between catechol and Fe^3+^ ions. By combining strong adhesion and tough cohesion, the BSA achieves adhesion strengths of 14.6 MPa at ambient temperature and 9.5 MPa at −196 °C, record‐high values among reported bio‐based adhesives as well as supramolecular adhesives to the best of our knowledge.^[^ ^17^, ^28^, ^31^, ^32^, ^37^, ^39^, ^52^, ^53^, ^54^, ^55^, ^56^, ^57^, ^58^ ^]^ Benefiting from its dynamic and reversible properties, it exhibits excellent multi‐reusability with more than 87% of the original adhesion strength remaining even after reused for ten times. The BSA also displays multi‐responsiveness to heat and near‐infrared light, broad applicability, and satisfactory resistance to aqueous solutions and common organic solvents. Therefore, an ultra‐strong and multi‐reusable adhesive from renewable feedstocks is successfully developed.

## 2 Result and Discussion

The preparation of BSA is described in Supporting Information. As shown in Figure S3, Supporting Information, the melevodopa functionalized castor oil is in a viscous state, and it turns into a stiff material after coordinating with Fe^3+^ ions, indicating that its cohesion is enhanced substantially. The formation of bis‐coordinated catechol‐Fe^3+^ bond in BSA was characterized by Raman spectroscopy. As shown in Figure S4, Supporting Information, characteristic peaks at 1273, 1317, 1424, and 1486 cm^−1^ are assigned to the catechol ring vibrations, and peaks at 589 and 636 cm^−1^ are assigned to the catechol‐Fe^3+^ bond vibrations.^[^ ^46^, ^49^ ^]^ The absence of characteristic peak at 528 cm^−1^ demonstrates that the main coordination state of catechol‐Fe^3+^ bond is bis‐ instead of tris‐state.^[^ ^47^ ^]^ The above results confirm the successful preparation of BSA through the construction of catechol‐Fe^3+^ bond.

**Figure 1:**
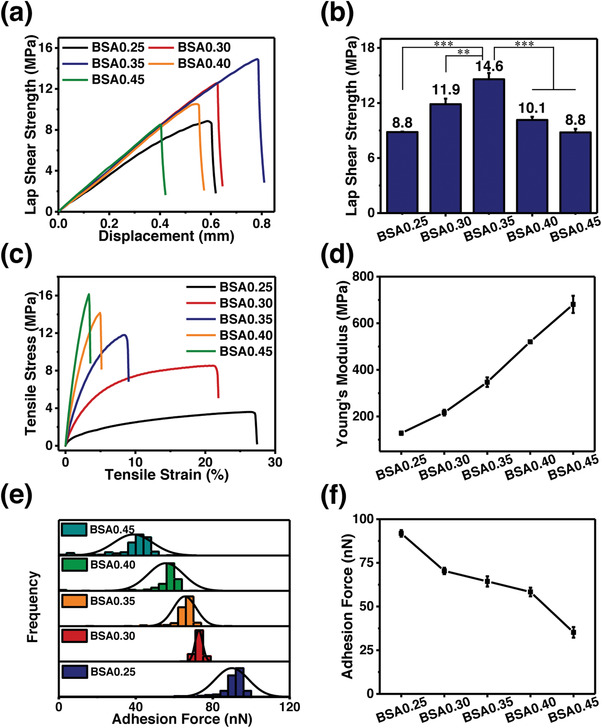
a) Lap shear strength‐displacement curves, b) lap shear strengths, (The sample size (n) was 3 for each data point. Data were expressed as mean ± standard deviation. Probability (*p*) values were determined by one‐way analysis of variance. Significance levels were indicated as *p < 0.05, **p < 0.01, and ***p < 0.001.) c) stress‐strain curves, d) Young's moduli, e) distribution of adhesion forces, and f) adhesion forces of BSA*x* films, where *x* changes from 0.25, to 0.30, 0.35, 0.40, and 0.45.

In our design, the molar ratio of Fe^3+^ ions and catechol units plays a significant role in the bonding strength of BSA. With the increase in Fe^3+^ ions, more catechol participates in forming coordination bonds, contributing to a high crosslinking degree and tough cohesion. However, this increase could result in weak adhesion due to less catechol interacting with adherends. In other words, the increase in Fe^3+^ ions affects cohesion and adhesion in an opposite way. To study the effect of Fe^3+^ ions content on the bonding strength of BSA, lap shear tests of BSA*x* bonded stainless steel sheets were performed (*x* indicates the molar ratio of Fe^3+^ ions and catechol units). As shown in **Figure** **1**a,b, by increasing *x* from 0.25 to 0.35, the bonding strength of BSA*x* increases from 8.8 to 14.6 MPa, respectively. This increase could be ascribed to the improvement of BSA's cohesion by adding more Fe^3+^ ions to coordinate with catechol. Meanwhile, by further increasing *x* from 0.35 to 0.45, the bonding strength of BSA*x* decreases from 14.6 to 8.8 MPa, respectively. The decrease could result from the decline of adhesion due to less uncoordinated catechol units. This result clearly shows that by partial coordination between melevodopa functionalized castor oil and Fe^3+^ ions, the BSA0.35 achieves a record‐high bonding strength of 14.6 MPa among reported bio‐based adhesives as well as supramolecular adhesives.^[^ ^17^, ^28^, ^31^, ^32^, ^37^, ^39^, ^52^, ^53^, ^54^, ^55^, ^56^, ^57^, ^58^ ^]^ The ultra‐high bonding strength can be represented by a visual demonstration. As shown in Figure S5 and Movie S1, Supporting Information, BSA0.35 bonded stainless steel sheets with a bonding area of 312.5 mm^2^ (12.5 mm × 25.0 mm) are capable of holding three adults with a total weight of ≈ 250 kg.

To investigate the reasons why the BSA0.35 achieves the highest bonding strength among BSA*x*, tensile tests and atomic force microscopy adhesion force analyses of BSA*x* films were performed. As shown in Figure 1c,d, by increasing *x* from 0.25 to 0.45, the Young's modulus of BSA*x* increases from 128 to 680 MPa, respectively, indicating a significant improvement in its cohesion. Compared with BSA0.25 and 0.30, the BSA0.35 exhibits a more robust mechanical strength and is capable of transmitting heavier loads among adherends without fracture, leading to higher bonding strength. As shown in Figure 1e,f, and Figures S7–S11, Supporting Information, by varying *x* from 0.25 to 0.45, the adhesion force of BSA*x* decreases from 92 to 35 nN, respectively, meaning a sharp decline in its adhesion. In comparison with BSA0.40 and 0.45, the BSA0.35 displays a stronger adhesion force and is capable of adhering to adherends more tightly, outperforming them in bonding. Both the BSA0.25 with the strongest adhesion force but the lowest Young's modulus and the BSA0.45 with the highest Young's modulus but the weakest adhesion force exhibit poorer adhesion performances than the BSA0.35. This result indicates that tough cohesion or strong adhesion is not the only factor critical to making an ultra‐strong bond, and only when adhesion and cohesion work together, can an ultra‐strong bond be created. Therefore, all of these results demonstrate that the BSA successfully achieves an ultra‐high bonding strength by combining tough cohesion with strong adhesion.

**Figure 2:**
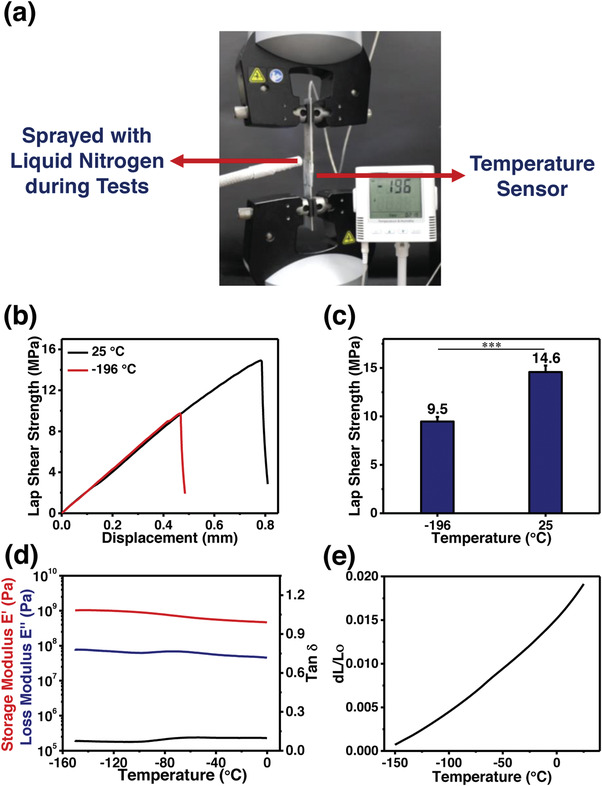
a) The digital image of lap shear tests performed at −196 °C. b) Lap shear strength‐displacement curves and c) lap shear strengths of BSA0.35 at 25 and −196 °C. (The sample size (n) was 3 for each data point. Data were expressed as mean ± standard deviation. Probability (*p*) values were determined by one‐way analysis of variance. Significance levels were indicated as *p < 0.05, **p < 0.01, and ***p < 0.001.) d) The DMA and e) thermal mechanical analysis results of BSA0.35 film at low‐temperature ranges.

Petroleum‐derived adhesives have found their advanced applications as cryogenic adhesives in space vehicles, medical, and research instruments, where they are required to display excellent adhesion performances at cryogenic temperature.^[^ ^38^, ^59^, ^60^ ^]^ We wonder whether the BSA could also realize a high adhesion strength at extremely low temperatures. To answer this question, lap shear tests of BSA0.35 bonded stainless steel sheets were performed at −196 °C. The bonded sheets were immersed in liquid nitrogen to lower their temperature to −196 °C prior to tests. In addition, as shown in **Figure** **2**a and Movie S2, Supporting Information, they were sprayed with liquid nitrogen during lap shear tests to keep them at −196 °C. As shown in Figure 2b,c, although there is a decline in its adhesion strengths, the BSA0.35 still achieves an adhesion strength of 9.5 MPa at −196 °C, a record‐high value among reported supramolecular adhesives to the best of our knowledge.^[^ ^17^, ^31^, ^32^, ^37^, ^38^, ^57^ ^]^ To investigate the reasons for the anti‐freezing, the dynamic mechanical analysis (DMA) test at low‐temperature ranges was performed. As shown in Figure 2d, there is no peak observed in the curve of tan *δ*, indicating that the BSA0.35 barely undergoes the physical transitions at low‐temperature ranges. Meanwhile, its storage modulus increases gradually with the decrease of the temperature. Both the stable structure and enhanced cohesion during cooling could contribute to achieving high adhesion strengths at cryogenic temperatures. However, as shown in Figure 2b, there is a decrease in bonding strengths by reducing the temperature to −196 °C, which could be due to stress concentrations developed during cooling. The main cause of stress concentrations is the difference in coefficients of thermal expansion between adhesive and adherends, which results in a volume change difference and the formation of stress on their interface. As shown in Figure 2e, the coefficient of thermal expansion of BSA0.35 is measured as 1.05 × 10^−4^ K^−1^, one order of magnitude higher than that of stainless steel. These results demonstrate that the BSA achieves a super‐high adhesion strength at −196 °C.

**Figure 3:**
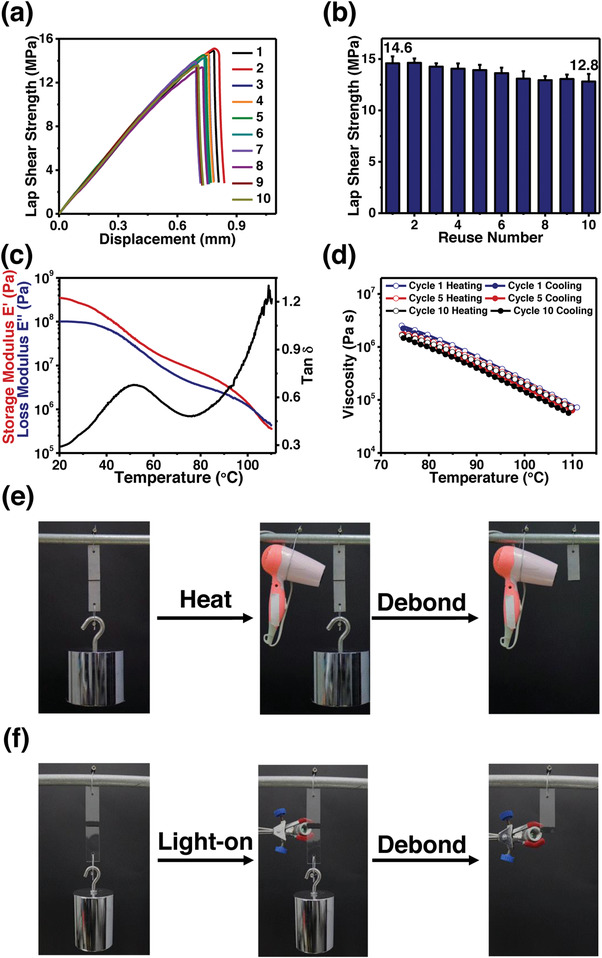
a) Lap shear strength‐displacement curves and b) lap shear strengths of BSA0.35 in bonding stainless steel after each reuse. c) The DMA result and d) cycle viscosity‐temperature curves of BSA0.35. e) Digital images of BSA0.35 bonded stainless steel sheets in responding to heat. f) Digital images of BSA0.35 bonded glass sheets in responding to near‐infrared light.

To investigate the reusability of BSA, lap shear tests of the rebonded stainless steel sheets were performed repeatedly. After regular tests, sheets were rebonded at original area with original adhesive films. As shown in **Figure** **3**a,b, the adhesion strength of BSA0.35 changes from 14.6 to 12.8 MPa, and more than 87% is reserved even after reuse for ten times. Unquestionably, the BSA0.35 displays excellent multi‐reusability, which could be attributed to its dynamic and reversible properties. To confirm this assumption, DMA, rheology, and cyclic tensile tests of BSA0.35 films were performed. As shown in Figure 3c, the storage modulus of BSA0.35 decreases from 3.5 × 10^8^ to 3.4 × 10^6^ Pa, and its loss modulus decreases from 1.0 × 10^8^ to 4.2 × 10^6^ Pa gradually by elevating the temperature from 20 to 110 °C. When the temperature reaches around 103 °C, its loss modulus exceeds the storage modulus, and the BSA0.35 changes from elastic to viscous states. A similar phenomenon was also observed in rheology tests. Figure 3d and Figure S12, Supporting Information, show that the viscosity of BSA0.35 decreases from 2.5 × 10^6^ to 7.2 × 10^4^ Pa s gradually by elevating the temperature from 75 to 110 °C, which could result from the disassociation of catechol‐Fe^3+^ bond and the rupture of supramolecular network. After reducing the temperature back to 75 °C, it returns to 2.2 × 10^6^ Pa s, which could be attributed to the reassociation of catechol‐Fe^3+^ bond and the reconstruction of supramolecular network. Such reversibility in the viscosity can be repeated ten times with negligible change. The cyclic tensile tests were performed to further study the reversible property. As shown in Figure S13, Supporting Information, the significant and overlapped hysteresis loops are observed in each cycles, which could result from the reversible disassociation and association of catechol‐Fe^3+^ bond. Based on these results, we could explain the reasons behind the excellent multi‐reusability of BSA0.35 as follows. With the increase in temperature, BSA0.35 films change from elastic to viscous states and could rewet the surface of adherends. Once cooled to ambient temperature, the BSA0.35 changes back to elastic state and could rebond adherends tightly. Such reversibility can be repeated ten times and therefore the BSA0.35 is endowed with excellent multi‐reusability.

The BSA also has the capability of responding to some external stimuli such as heat and near‐infrared light. Figure 3e and Movie S3, Supporting Information, show that BSA0.35 bonded stainless steel sheets hanging a 10 kg weight are separated after being heated by a hair dryer for ≈ 1 min. In addition, the BSA is capable of responding to near‐infrared light owing to the photothermal effect of catechol‐Fe^3+^.^[^ ^61^, ^62^ ^]^ As shown in Figure 3f, Figures S14 and S15, and Movie S4, Supporting Information, BSA0.35 bonded glass sheets easily sustain a 4 kg weight and they are separated once exposed to an 808 nm near‐infrared light with a power density of 2 W cm^−2^ for ≈ 4 min. In other words, the BSA displays excellent multi‐reusability and multi‐responsiveness.

**Figure 4:**
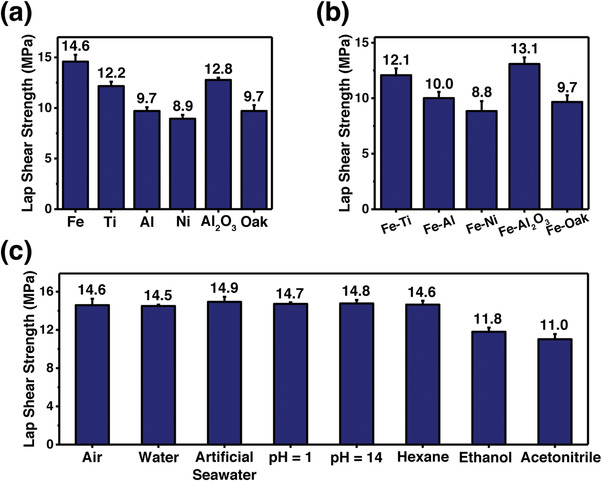
a) Lap shear strengths of BSA0.35 in bonding stainless steel (Fe), titanium alloy (Ti), aluminum alloy (Al), nickel (Ni), aluminum oxide (Al_2_O_3_), and oak wood (Oak). b) Lap shear strengths of BSA0.35 in bonding Fe with Ti, Al, Ni, Al_2_O_3_, and Oak. c) Lap shear strengths of BSA0.35 in bonding Fe after being soaked in different kinds of solvents for 24 h.

To assess the applicability of BSA, lap shear tests of BSA0.35 bonded sheets made of different materials were performed. As shown in **Figure** **4**a and Figure S16a, Supporting Information, the BSA0.35 is capable of bonding a wide range of metal materials tightly and adhesion strengths in bonding stainless steel, titanium alloy, aluminum alloy, and nickel are as high as 14.6, 12.2, 9.7, and 8.9 MPa, respectively. For non‐metal materials such as ceramics and natural materials, the BSA0.35 shows outstanding performance in bonding aluminum oxide and oak wood, reaching 12.8 and 9.7 MPa, respectively. The BSA0.35 also performs well in bonding two different adherends. Figure 4b and Figure S16b, Supporting Information, show that adhesion strengths of BSA0.35 in bonding stainless steel with titanium alloy, aluminum alloy, nickel, aluminum oxide, and oak wood are as high as 12.1, 10.0, 8.8, 13.1, and 9.7 MPa, respectively. The wide applicability of BSA could be attributed to the catechol unit that can interact with different materials through hydrogen bonding, metal‐ligand coordination, hydrophobic effect, etc.^[^ ^26^, ^44^, ^45^ ^]^ Therefore, the BSA exhibits broad applicability in bonding not only the same but also different materials.

A good adhesive must work well under various service conditions such as underwater environments. To ascertain the solvent resistance of BSA, lap shear tests of BSA0.35 bonded stainless steel sheets were performed after being soaked in various solvents for 24 h. As shown in Figure 4c and Figure S16c, Supporting Information, adhesion strengths of BSA0.35 are almost reserved after being soaked in water, artificial seawater, acid solution (pH = 1), and alkaline solution (pH = 14), achieving 14.5, 14.9, 14.7, and 14.8 MPa, respectively. Apart from aqueous solutions, the resistance of BSA to common organic solvents was also evaluated. For organic solvents with relatively low polarity such as hexane, the BSA0.35 maintains its ultra‐high bonding strength of 14.6 MPa. For organic solvents with relatively high polarity such as ethanol and acetonitrile, the BSA0.35 still reaches more than 80% and 75% of its original adhesion strength, respectively. The satisfactory solvent resistance of BSA could be attributed to the strong bonding strength of catechol‐Fe^3+^, along with its hydrophobic nature.^[^ ^50^ ^]^ All of the results confirm that the BSA displays another excellent service performance that can be resistant to aqueous solutions and common organic solvents.

**Figure 5:**
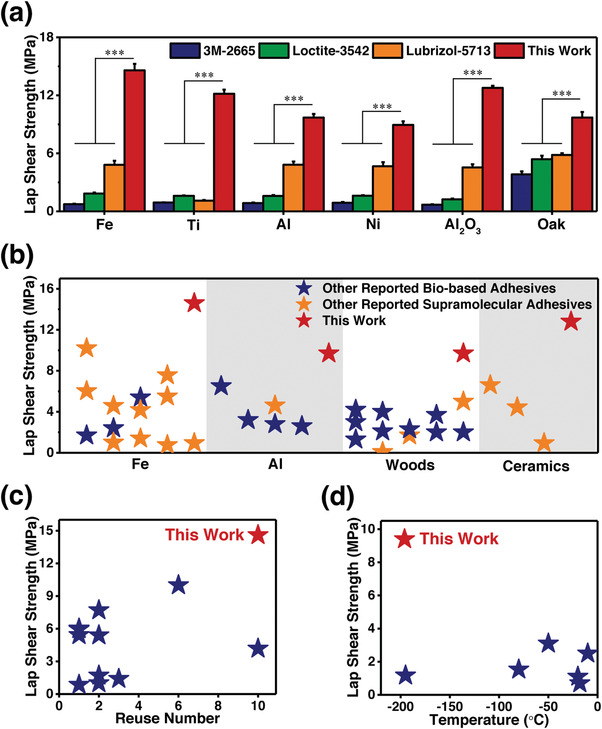
a) The comparison of BSA with common commercial petroleum‐derived adhesives in bonding Fe, Ti, Al, Ni, Al_2_O_3_, and Oak. (The sample size (n) was 3 for each data point. Data were expressed as mean ± standard deviation. Probability (*p*) values were determined by one‐way analysis of variance. Significance levels were indicated as *p < 0.05, **p < 0.01, and ***p < 0.001.) b) The comparison of BSA with reported bio‐based adhesives as well as supramolecular adhesives in bonding Fe, Al, woods, and ceramics.^[^ ^14^, ^17^, ^19^, ^28^, ^31^, ^32^, ^37^, ^39^, ^52^, ^53^, ^54^, ^55^, ^56^, ^57^, ^58^, ^63^, ^64^, ^65^, ^66^, ^67^, ^68^, ^69^, ^70^, ^71^ ^]^ c) The comparison of BSA with reported supramolecular adhesives in adhesion strengths and reuse numbers.^[^ ^17^, ^28^, ^31^, ^32^, ^37^, ^39^, ^52^, ^55^, ^56^, ^57^ ^]^ d) The comparison of BSA with reported supramolecular adhesives in adhesion strengths at low temperature.^[^ ^17^, ^31^, ^32^, ^37^, ^38^, ^57^ ^]^

A comparison between the BSA and common commercial petroleum‐derived adhesives was made to demonstrate its outstanding adhesion performances. As shown in **Figure** **5**a and Figure S16d–f, Supporting Information, the BSA presents the highest adhesion strengths among 3M‐2665, Loctite‐3542, and Lubrizol‐5713 in bonding different materials. Not only petroleum‐derived adhesives, a comprehensive comparison between the BSA and reported bio‐based adhesives, as well as supramolecular adhesives, was also made. As shown in Figure 5b, the BSA outperforms reported bio‐based adhesives and supramolecular adhesives in bonding stainless steel, aluminum alloy, woods, and ceramics to the best of our knowledge.^[^ ^14^, ^17^, ^19^, ^28^, ^31^, ^32^, ^37^, ^39^, ^52^, ^53^, ^54^, ^55^, ^56^, ^57^, ^58^, ^63^, ^64^, ^65^, ^66^, ^67^, ^68^, ^69^, ^70^, ^71^ ^]^ In addition, we made another two comprehensive comparisons between the BSA and reported supramolecular adhesives to further demonstrate its robust bonding strength, excellent multi‐reusability, and outstanding performances at low temperature. Figure 5c shows that the BSA exhibits the highest adhesion strength and the largest reuse number at the same time.^[^ ^17^, ^28^, ^31^, ^32^, ^37^, ^39^, ^52^, ^55^, ^56^, ^57^ ^]^ Meanwhile, as shown in Figure 5d, the BSA presents the highest bonding strength at the lowest temperature.^[^ ^17^, ^31^, ^32^, ^37^, ^38^, ^57^ ^]^

## 3 Conclusion

In summary, we have reported an ultra‐strong and multi‐reusable BSA through partial coordination between melevodopa functionalized castor oil and Fe^3+^ ions. By combining strong adhesion with tough cohesion, the prepared BSA achieves record‐high adhesion strengths of 14.6 MPa at ambient temperature and 9.5 MPa at −196 °C. Benefiting from its dynamic and reversible properties, the BSA exhibits excellent multi‐reusability with more than 87% adhesion strength remaining even after reuse for ten times. Moreover, it displays multi‐responsiveness to heat and near‐infrared light, wide applicability, and satisfactory solvent resistance. In a word, the BSA presents robust adhesion performances for practical and advanced applications. With the increasing and urgent need to develop environment‐friendly and sustainable adhesives, it is highly anticipated that the present research will provide a novel strategy for high‐performance and reusable adhesives sourced from renewable feedstocks.

## Conflict of Interest

The authors declare no conflict of interest.

## Supporting information

Supporting Information

Supporting Movie 1

Supporting Movie 2

Supporting Movie 3

Supporting Movie 4

Supporting Movie 5

## Data Availability

The data that support the findings of this study are available from the corresponding author upon reasonable request.
