# Supplementary material for: A Bio‐Based Supramolecular Adhesive: Ultra‐High Adhesion Strengths at both Ambient and Cryogenic Temperatures and Excellent Multi‐Reusability
Source: Adv Sci (Weinh). 2022 Aug 9;9(28):2203182. doi: 10.1002/advs.202203182 (PMC9534982; doi:10.1002/advs.202203182)
Supplement: Supplementary file 1 — Supporting Information [file ADVS-9-2203182-s004.pdf]

ADVANCED SCIENCE
Open Access

Supporting Information

for *Adv. Sci.*, DOI 10.1002/advs.202203182

A Bio-Based Supramolecular Adhesive: Ultra-High Adhesion Strengths at both Ambient and Cryogenic Temperatures and Excellent Multi-Reusability

Peng Sun, Shan Mei, Jiang-Fei Xu* and Xi Zhang*

Supporting Information

A Bio-Based Supramolecular Adhesive: Ultra-High Adhesion Strengths at Both Ambient and Cryogenic Temperatures and Excellent Multi-Reusability

Peng Sun, Shan Mei, Jiang-Fei Xu* and Xi Zhang*

Key Laboratory of Organic Optoelectronics & Molecular Engineering, Department of Chemistry, Tsinghua University, Beijing 100084, China.

* corresponding author: pengsun@mail.tsinghua.edu.cn; xujf@mail.tsinghua.edu.cn

Experimental

Materials

Castor oil was purchased from Alfa Aesar. L-3,4-Dihydroxyphenylalanine methyl ester hydrochloride was purchased from Hao Hong Pharma. *N,N'*-Disuccinimidyl carbonate, ferric chloride ($FeCl_3$), trimethylamine (TEA), dry acetonitrile (ACN), dry dichloromethane (DCM), and dry pyridine were purchased form J&K. Chloroform, DCM, ethyl acetate (EA), petroleum ether (PE), methanol (MA), sodium chloride (NaCl), and sodium sulphate (Na_2SO_4) were purchased from Bei Jing Tong Guang Fine Chemicals Company and used as received. Castor oil was purified by a silica column with PE/EA (v/v = 5/1) as the eluent to remove the impurity.

Characterization

^{1}H-NMR spectra were recorded on a JOEL JNM-ECA400 spectrometer (400 MHz) at room temperature using dimethyl sulfoxide (DMSO-d_6) as the solvent.

ESI-MS spectra were recorded on a SHIMADZU LCMS-IT/TOF system in a positive mode using methanol as the mobile phase.

Raman spectra were recorded on a LabRAM HR Evolution machine. The 532 nm laser excitation was used in combination with a 50× microscope objective. The spectra were acquired using an air-cooled charge coupled device behind a grating (600 g·mm^{-1}) spectrograph from 450 to 1700 cm^{-1}. For the Raman spectrum of the melevodopa functionalized castor oil, the laser power was 13 mW/cm^2 and that of 0.325 mW/cm^2 in collecting the Raman spectrum of the bio-based supramolecular adhesive (BSA). The exposure time was 60 s and both Raman spectra consisted of 1 accumulation.

Both the BSA and commercial petroleum-derived adhesives were melted into films by using a ZB-910G table press under 110 °C and 15 MPa within 10 minutes.

Lap shear tests were conducted by using a INSTRON 68TM-5 universal testing machine with a strain rate of 10 mm/min at room temperature or at -196 °C. Adhesive films were cut into rectangle shapes (25 mm × 12.5 mm × 0.2 mm) and placed between two identical substrates (100 mm × 25 mm × 1.6 mm) with an overlap area of 312.5 mm^2 (25 mm × 12.5 mm). In particular, the thickness of the aluminium oxide sheet changed into 4.0 mm due to its brittle nature. Two paper clips held substrates together and two stainless steel wires with a thickness of 0.2 mm were used to control the thickness of the adhesive while heating. The thermal treatment was performed in an oven at 100 °C for 5 min. Prior to the lap shear test, the bonded sheets were cooled down to the room temperature and stood for another 24 hours. Particularly, for lap shear tests at -196 °C, the bonded sheets were immersed in liquid nitrogen prior to tests to lower the temperature to -196 °C and they were also sprayed with liquid nitrogen during tests to keep them at -196 °C.

The stress-strain curves were measured by using a INSTRON 68TM-5 universal testing machine with a strain rate of 50 mm/min at room temperature. Samples were cut into a rectangle shape (60 mm × 10 mm × 0.2 mm) and the gauge length was around 25 mm. The Young's modulus was calculated as the initial slope of the linear stage of the stress-strain curve. Cyclic tensile tests were performed at a strain of 4% without the rest.

The adhesion force was measured on a Cypher VRS atomic force microscopy (AFM) machine utilizing Oxford model AC240TS-R3 tips at room temperature. Igor Pro 6.37 data analysis software was used to process images and analyze force curves. To obtain the adhesion force of each BSA*x*, the force mapping mode study was performed, which was based on recording the force-displacement curves of 256 spots at a random area of BSA*x* films. As illustrated in Figure S6, for each spot, the value of the adhesion force is taken as the difference between the horizontal and minimum values on the retraction curves. For each measurement, the scanning area was fixed at 400 μm^2 (20 μm × 20 μm) and force-displacement curves were

recorded with a rate of 0.5 or 1.0 Hz. For each BSA*x*, the value of the adhesion force was taken as the average value of that of 256 spots and the measurement was repeated for at least 3 times.

The thermal mechanical analysis (TMA) was conducted on a TMA 402 F1/F3 Hyperion machine. Rectangular samples (20 mm × 7 mm × 0.2 mm) were heated from -150 to 25 °C with a heating rate of 5 °C/min and a static force of 0.05 N. The mean linear thermal coefficient of expansion was measured as the slope between endpoints of the curve.

The dynamic mechanical analysis (DMA) was conducted on a TA instrument Discovery 850 machine. Rectangular samples (30 mm × 7 mm × 0.2 mm) were heated from 20 to 110 °C or from -150 to 0 °C with a heating rate of 5 °C/min and a constant frequency of 1 Hz.

Rheology tests were performed on a TA Discovery HR-2 rheometer using the parallel plate geometry (25 mm diameter). Samples were heated from 75 to 110 °C with a rate of 5 °C/min and cooled from 110 to 75 °C with the same rate. A constant strain of 0.1% and a constant angular frequency of 0.5 rad/s were used.

Preparation of melevodopa functionalized castor oil.

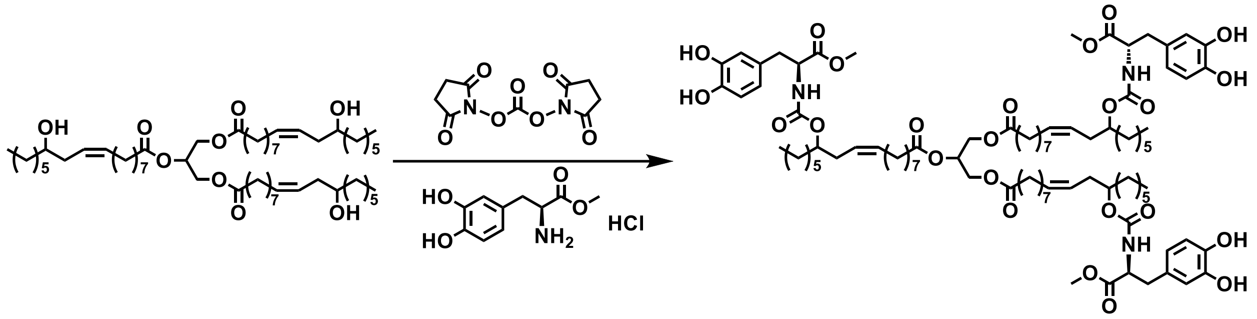

Castor oil (14.0 g, 15.0 mmol) and *N,N'*-disuccinimidyl carbonate (19.2 g, 75.0 mmol) were dissolved by dry DCM (50 mL) and dry ACN (150 mL), which were added with dry pyridine (4.7 g, 59.4 mmol). After reacting for 24 hours at 50 °C, the mixture was concentrated, diluted by DCM (50 mL), and filtrated to remove the unreacted *N,N'*-disuccinimidyl carbonate. After being concentrated, the mixture was distilled at 70 °C to remove pyridine. Then the mixture was diluted by DCM (300 mL) and washed by brine (3 x 200 mL). The collected

organic phase was dried by Na_2SO_4, filtrated, and concentrated. Then the resultant sticky solid was dissolved by chloroform (60 mL), and added with L-3,4-dihydroxyphenylalanine methyl ester hydrochloride (11.8 g, 48 mmol) and TEA (5.2 g, 54 mmol). The mixture was stirred at room temperature for 4 hours and then concentrated. The resultant sticky solid was dissolved by DCM (300 mL) and washed by brine (3 x 200 mL). The organic phase was collected and dried by Na_2SO_4. After being filtrated and concentrated, the resultant crude product was purified by a silica column with DCM/MA (v/v = 100/3) as the eluent to afford the melevodopa functionalized castor oil (8.0 g, 32.4%). ^{1}H-NMR (DMSO), δ (ppm): 8.71 (d, 6H), 7.37 (d, 3H), 6.58 (d, 6H), 6.45 (d, 3H), 5.32 (m, 7H), 4.53 (m, 3H), 4.23 (m, 2H), 4.11 (m, 5H), 3.58 (s, 9H), 2.79 (m, 6H), 2.27 (m, 12H), 1.98 (m, 6H), 1.40 (m, 60H), 0.84 (t, 9H). ESI-MS (m/z): calculated for $[M+Na]^+$ = 1666.9484, found $[M+Na]^+$ = 1666.9484.

Preparation of BSA*x*.

The BSA0.35 was prepared by the following steps. The melevodopa functionalized castor oil (1 g, 0.6 mmol) was dissolved by MA (6 mL). Then the mixture was added with $FeCl_3$ (0.103 g, 0.64 mmol) and TEA (0.368 g, 3.6 mmol). After standing at room temperature for 12 hours, the resultant gel was dried under vacuum at 50 °C for 6 hours to remove MA and TEA, getting 1.05 g BSA0.35 with a yield of 95%. The BSA0.25, 0.30, 0.40, and 0.45 were prepared through the similar procedures but with a different dosage of $FeCl_3$.

Multi-reuse experiments.

After usual lap shear tests, the separated stainless steel sheets with fractured BSA0.35 films remained were clamped together again and placed in an oven at 100 °C for 5 min. After standing at room temperature for 24 hours, the second adhesion strength of BSA0.35 was recorded through lap shear tests. The above bonding-testing procedures were repeated for another eight times to get the whole ten adhesion strengths of BSA0.35.

Solvent resistance experiments.

Solvent resistance experiments were performed by soaking BSA0.35 bonded stainless steel specimens in different kinds of solvents, including water, artificial sea water, acid solution (pH = 1), alkaline solution (pH = 14), hexane, ethanol, and acetonitrile for 24 hours at room temperature. Lap shear tests were conducted immediately without any treatment.

Statistical Analysis

The data obtained was used without pre-processing unless noted otherwise. Data was expressed as mean ± standard deviation. The sample size (n) was 3 for each data point. Probability (p) values were determined by one-way analysis of variance using Microsoft Excel. Significance levels were indicated as $^*p < 0.05$, $^{**}p < 0.01$, and $^{***}p < 0.001$.

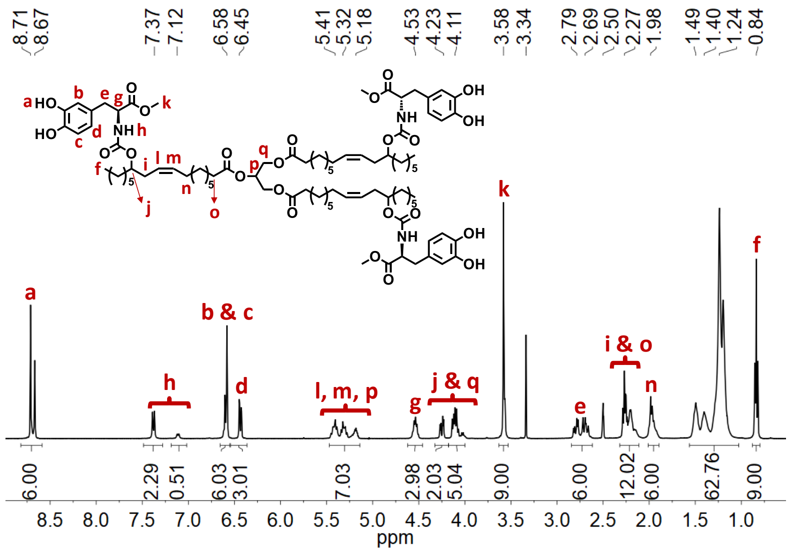

Figure S1. The ^{1}H-NMR spectrum of melevodopa functionalized castor oil.

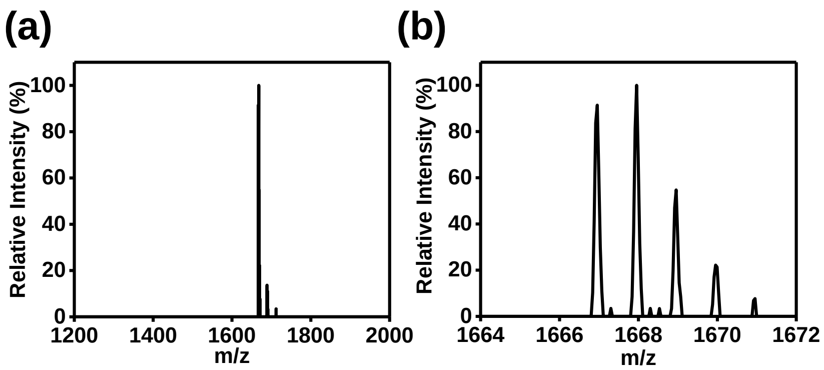

Figure S2. (a) Full and (b) expanded ESI-MS spectra of melevodopa functionalized castor oil.

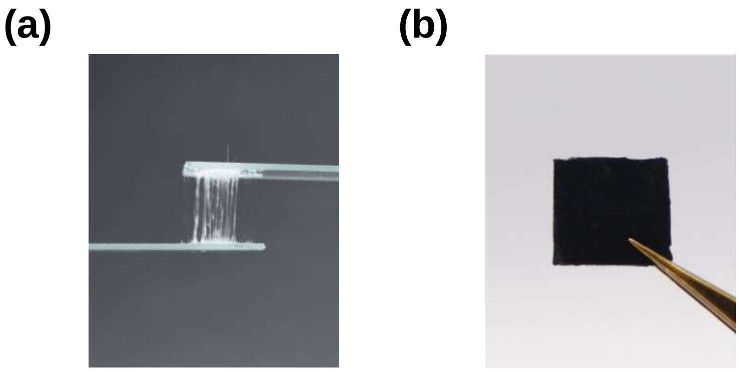

Figure S3. The digital images of (a) melevodopa functionalized castor oil and (b) BSA0.35.

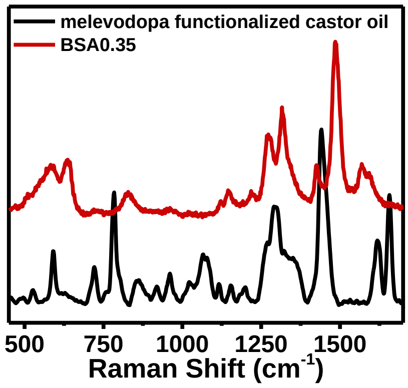

Figure S4. Raman spectra of melevodopa functionalized castor oil and BSA0.35.

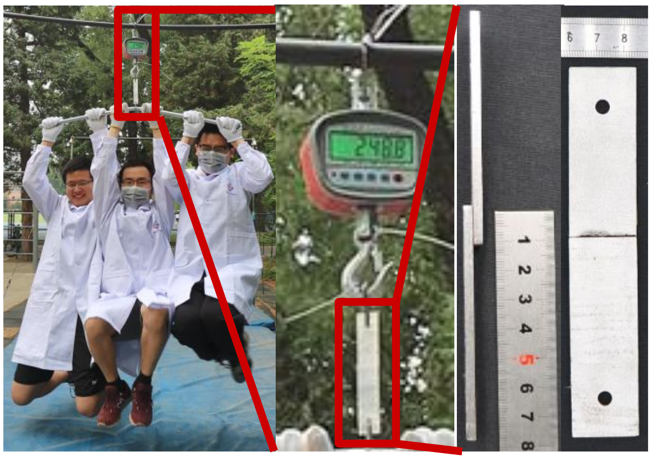

Figure S5. The digital image of BSA0.35 bonded stainless steel sheets to lift three adults with a total weight of ~ 250 kg with a bonding area of 312.5 mm^2.

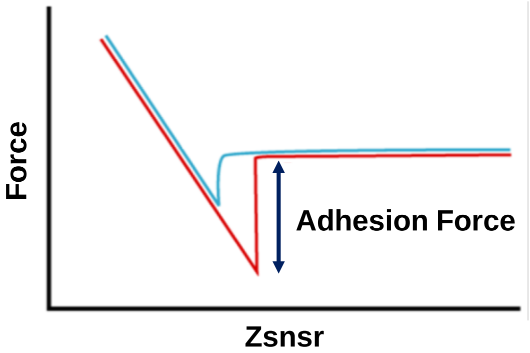

Figure S6. The schematic diagram of the measurement of the adhesion force from the force-displacement curve. The red line is the retraction curve. The x axis, ZSnsr (Z sensor), represents the displacement between the sample surface and the resting position of the cantilever (rather than the actual distance between the sample surface and the AFM tip).

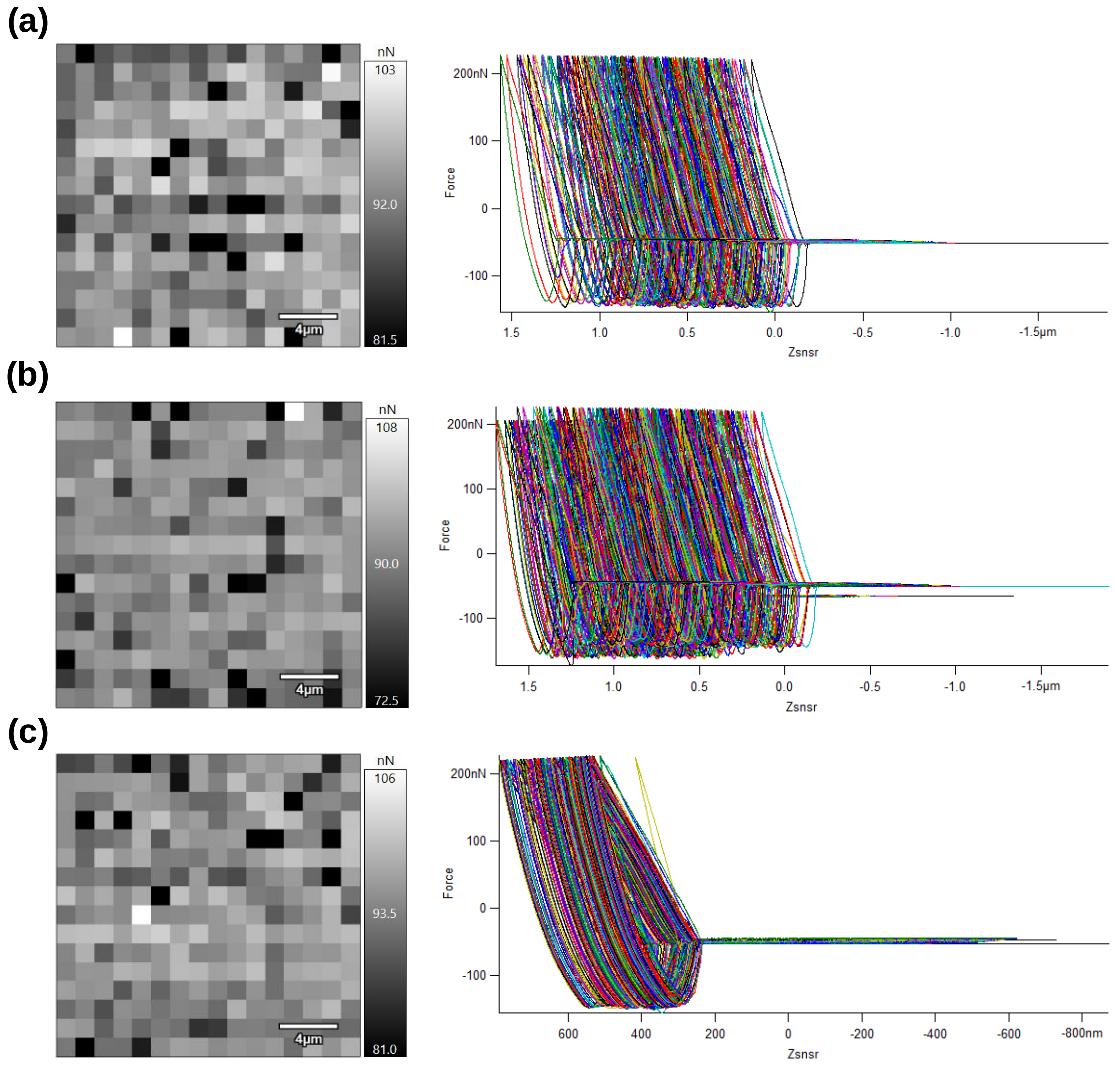

Figure S7. Force maps and corresponding force-displacement curves of BSA0.25 samples.

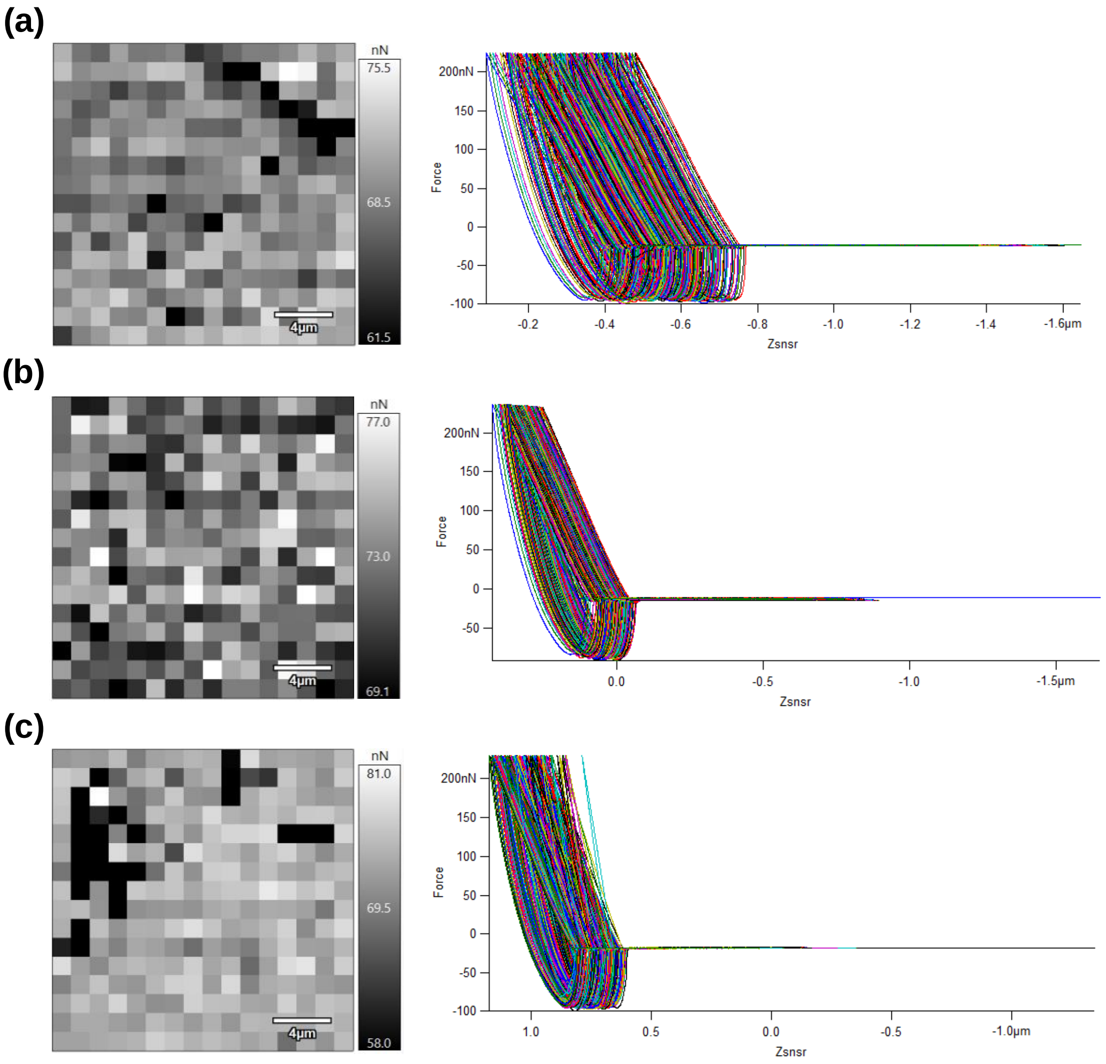

Figure S8. Force maps and corresponding force-displacement curves of BSA0.30 samples.

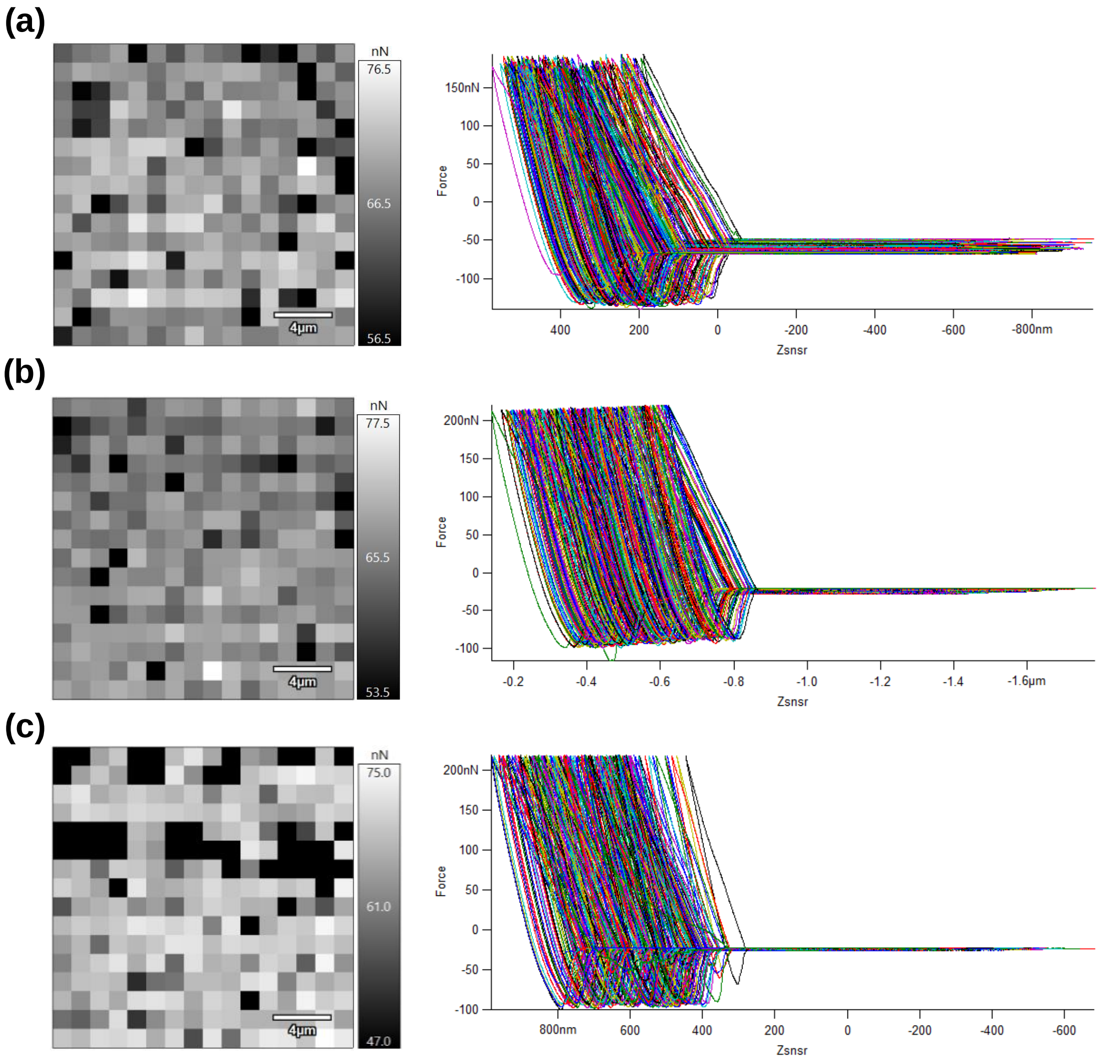

Figure S9. Force maps and corresponding force-displacement curves of BSA0.35 samples.

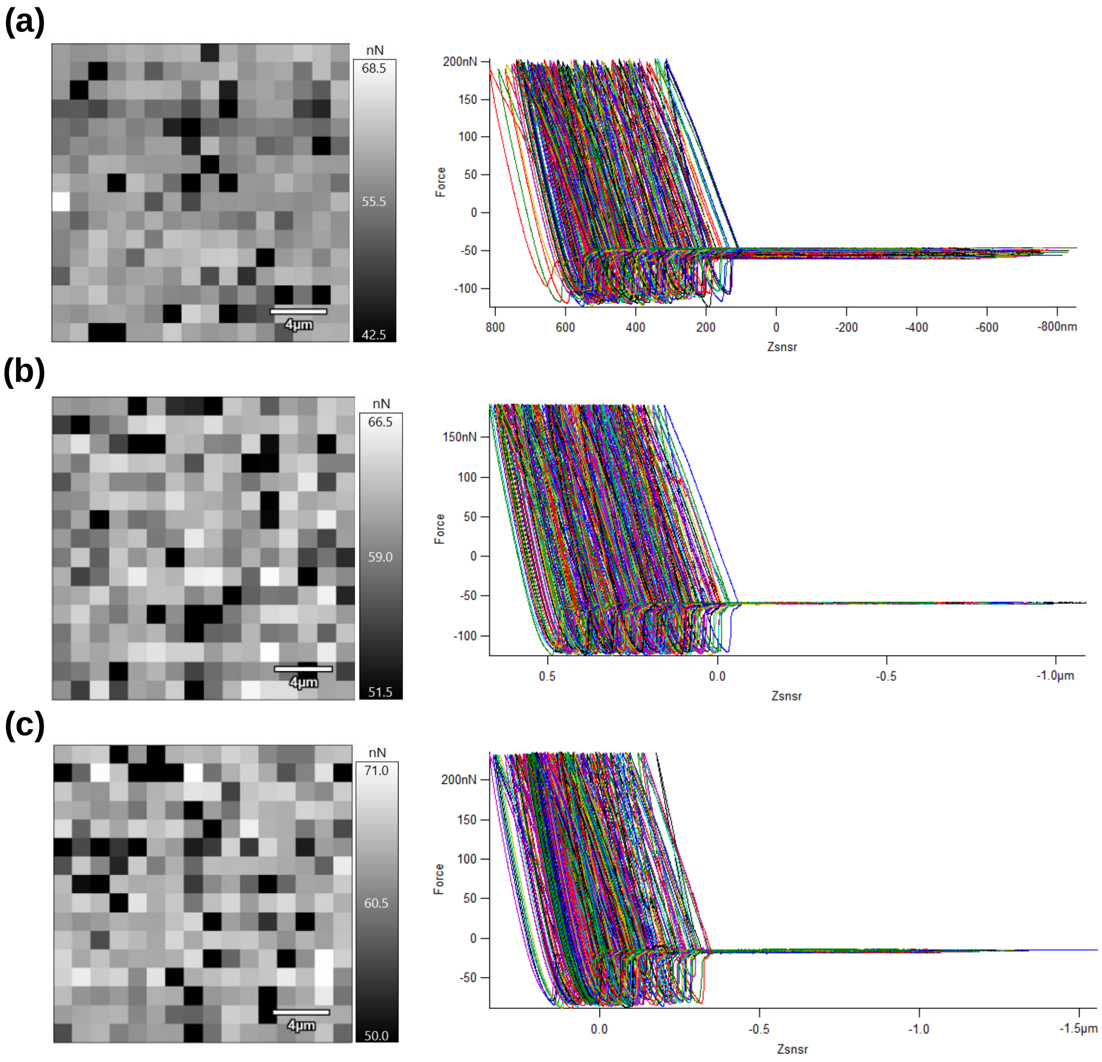

Figure S10. Force maps and corresponding force-displacement curves of BSA0.40 samples.

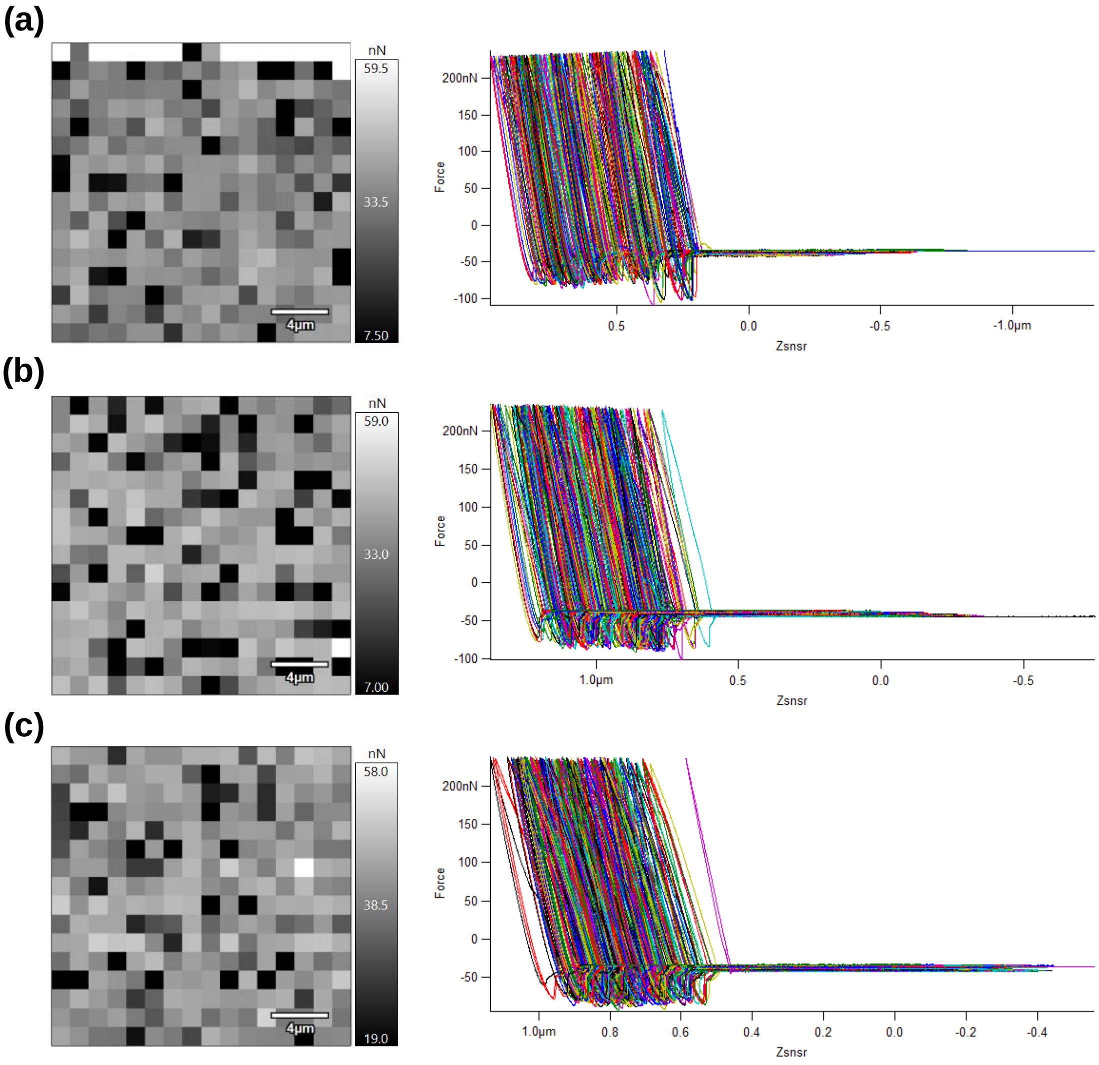

Figure S11. Force maps and corresponding force-displacement curves of BSA0.45 samples.

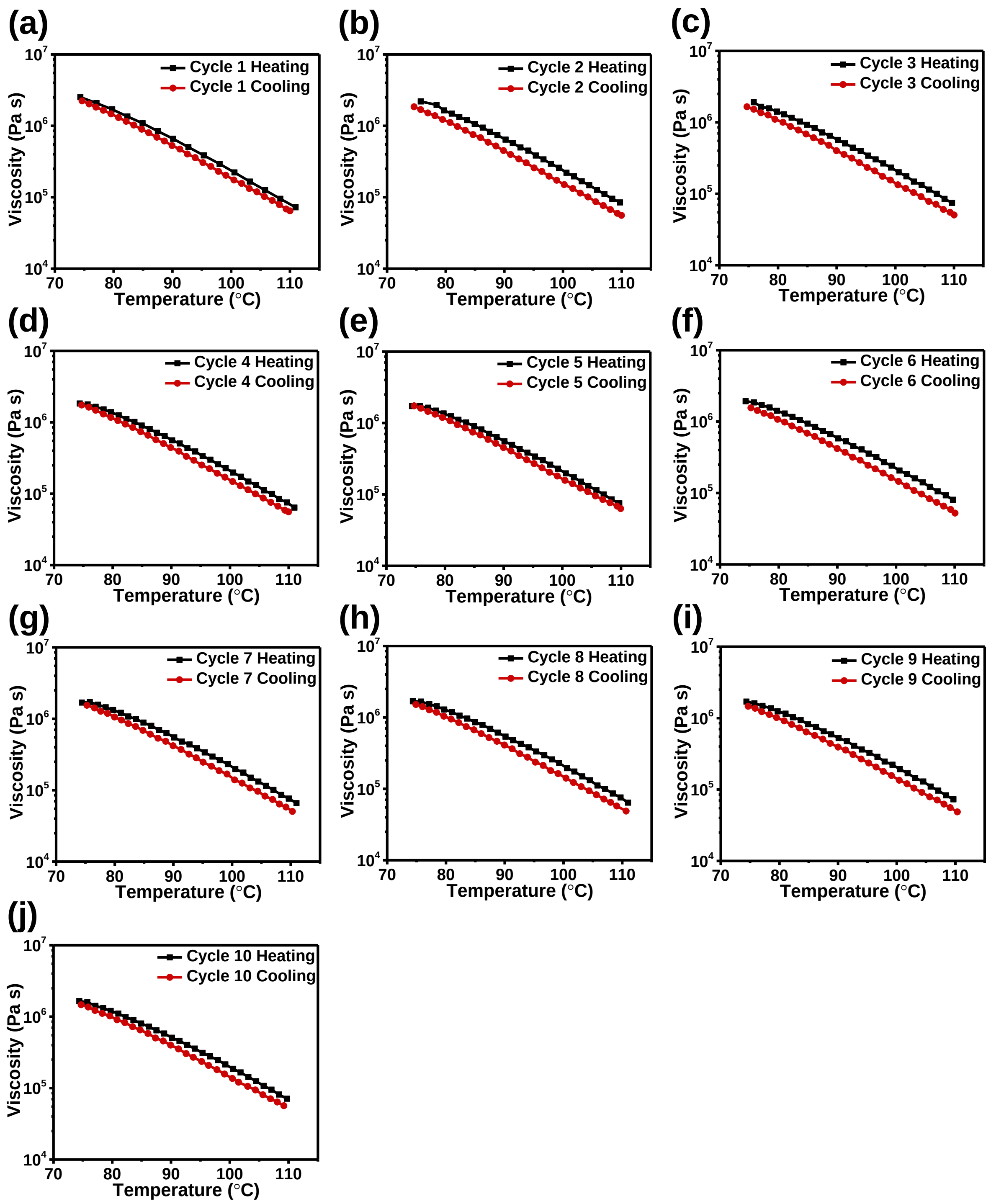

Figure S12. Viscosity-temperature curves of BSA0.35 in each cycle.

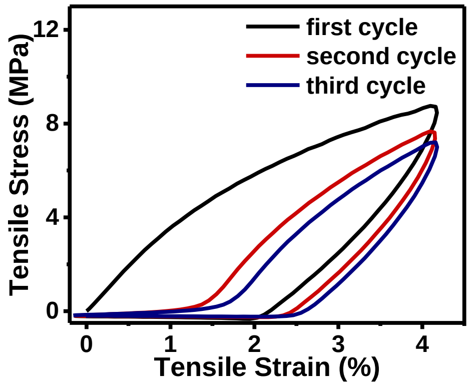

Figure S13. Cyclic tensile test curves of BSA0.35.

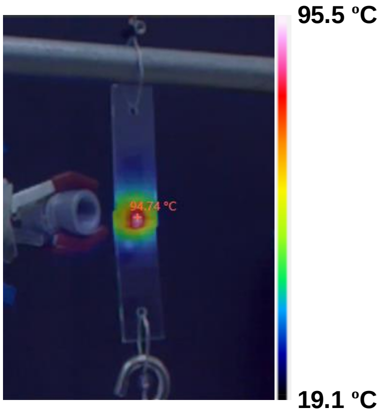

Figure S14. The digital image of BSA0.35 bonded glass sheets exposed to an 808 nm near-infrared light with a power density of 2 W/cm^2.

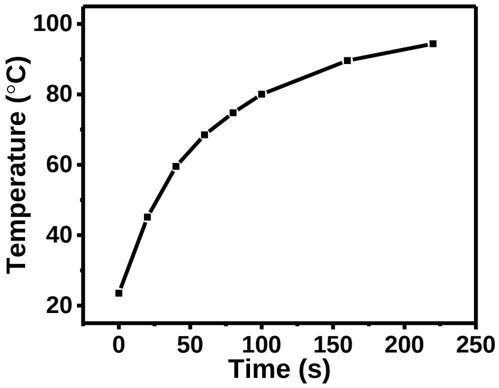

Figure S15. The temperature-time curve of BSA0.35 bonded glass sheets exposed to an 808 nm near-infrared light with a power density of 2 W/cm^2.

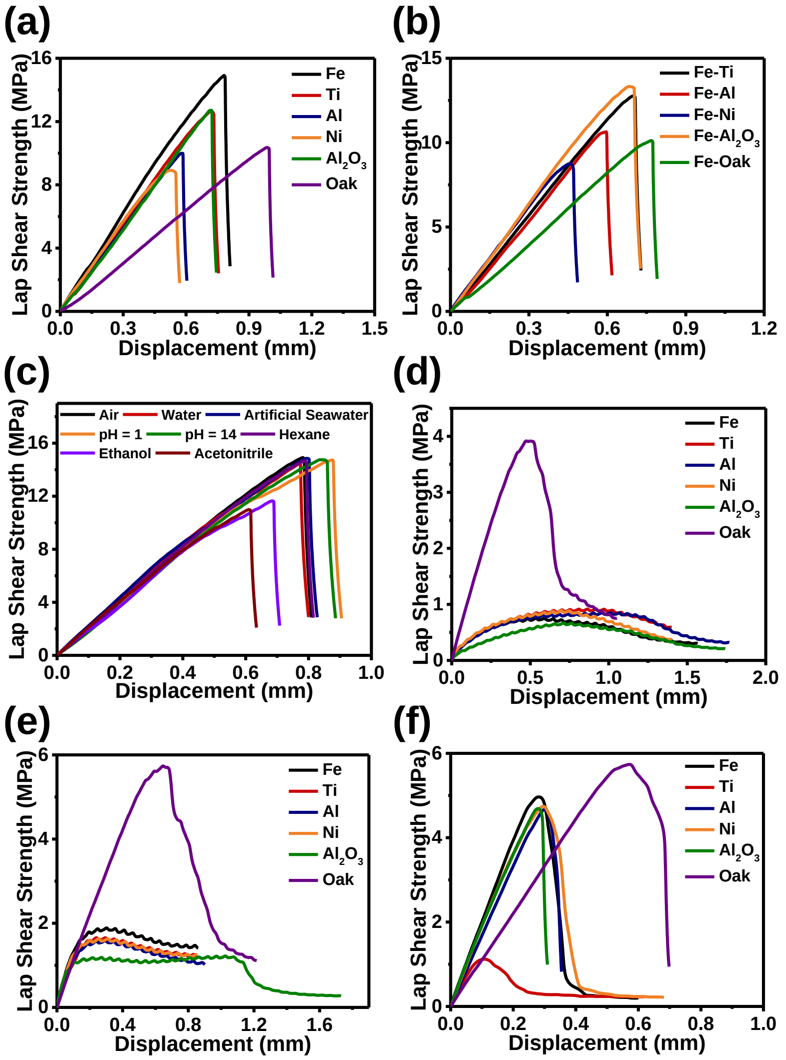

Figure S16. (a) Lap shear strength-displacement curves of BSA0.35 in bonding stainless steel (Fe), titanium alloy (Ti), aluminium alloy (Al), nickel (Ni), aluminium oxide (Al_2O_3), and oak wood (Oak). (b) Lap shear strength-displacement curves of BSA0.35 in bonding Fe with Ti, Al, Ni, Al_2O_3, and Oak. (c) Lap shear strength-displacement curves of BSA0.35 in bonding Fe after being soaked in water, artificial seawater, acid solution (pH = 1), alkaline solution (pH = 14), hexane, ethanol, and acetonitrile for 24 hours. (d) Lap shear strength-displacement curves of 3M-2665 in bonding Fe, Al, Ni, Ti, Al_2O_3, and Oak. (e) Lap shear strength-displacement curves of Loctite-3542 in bonding Fe, Al, Ni, Ti, Al_2O_3, and Oak. (f) Lap shear strength-displacement curves of Lubrizol-5713 in bonding Fe, Al, Ni, Ti, Al_2O_3, and Oak.

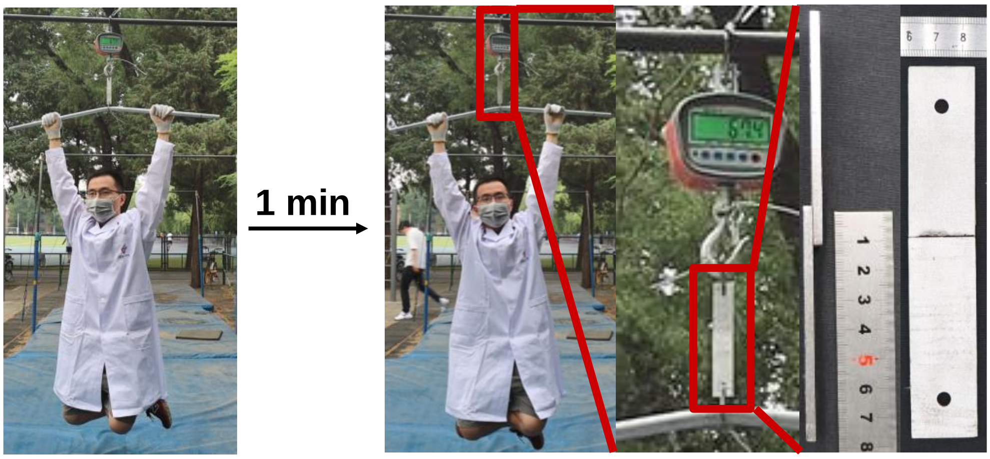

Figure S17. The digital image of BSA0.35 bonded stainless steel sheets to lift an adult with a weight of 67 kg with a bonding area of 312.5 mm^2.
